# Importance of ultradian oscillations in neurogenesis during development and its implications for spinal cord regeneration

**DOI:** 10.3389/fcell.2025.1680322

**Published:** 2025-10-08

**Authors:** Sami A. Leino, Ximena Soto

**Affiliations:** Division of Molecular and Cellular Function, School of Biological Sciences, Faculty of Biology, Medicine and Health, The University of Manchester, Manchester, United Kingdom

**Keywords:** oscillations, neurogenesis, regeneration, development, microRNA, BHLH factors

## Abstract

The formation of a functional nervous system during development and its maintenance in adulthood rely on precise regulation of neural stem cell (NSC) proliferation and differentiation. During neurogenesis, progenitor cells use various cellular and molecular mechanisms to balance these processes. Among these, dynamic signal encoding, specifically ultradian oscillations, which are regular protein fluctuations occurring over a few hours, has emerged as a key mechanism underlying NSC fate decisions. In adults, reactivation of quiescent NSCs, proliferation, and differentiation are also controlled by ultradian oscillations. Furthermore, these ultradian dynamics signals are modulated by microRNAs and are considered critical for the ability of neural progenitors to transition between different states. Altogether, these findings may have potential significance for our understanding of NSC reactivation and differentiation in the context of injury or neurodegeneration. The mammalian spinal cord harbours endogenous multipotent NSCs that respond to injury but mostly generate astrocytes and do not undergo neurogenesis. By contrast, many anamniotes regenerate spinal cord neurons from endogenous progenitors, despite the same molecular signalling pathways being activated, suggesting that subtle differences in how these pathways are regulated may result in different regenerative outcomes. Whether oscillatory dynamics could influence the reactivation and differentiation of NSCs upon spinal cord injury remains to be determined. This review explores the role of transcription factor ultradian oscillations in neurogenesis and how microRNAs modulate them. Additionally, we examine evidence for the role of ultradian dynamics in the reactivation of quiescent NSCs and their potential significance for regenerative neurogenesis in the context of spinal cord injury.

## 1 Introduction

The embryonic development of the central nervous system (CNS) is a complex multi-step process that is characterised by the formation of the neural tube, neurogenesis, and gliogenesis, which are critical for establishing the structure and function of the CNS. This process begins with the symmetric division of neuroepithelial cells, the first type of neural stem cell (NSC), to expand the NSC population. These cells then undergo a morphological transformation into radial glial cells (RGCs). Subsequently, RGCs begin to divide asymmetrically, initially producing neurons and later generating glial cell types, including oligodendrocytes, ependymal cells, and astrocytes (Kageyama et al., 2015; Paridaen and Huttner, 2014; Imayoshi and Kageyama, 2014). While NSCs persist in the adult brain and support ongoing neurogenesis in localised regions of the CNS (Alvarez-Buylla et al., 2001), adult mammalian NSCs normally stay in a non-proliferative and undifferentiated state known as quiescence, infrequently transitioning to an active state to produce new neurons, thus having a limited overall cellular turnover (Kaise and Kageyama, 2024; Reynolds and Weiss, 1992; Doetsch et al., 1997; Zhou et al., 2022). Adult NSCs can be activated under pathological conditions, such as spinal cord injury. In mammals, which have limited regenerative capacity, they primarily differentiate into astrocytes. By contrast, in regenerative species such as zebrafish (Reimer et al., 2008) and salamanders (Tazaki et al., 2017), NSCs differentiate into neurons upon injury. In these species, radial glial cells within the spinal ependymal zone can undergo *de novo* neurogenesis to repair the spinal cord. Equivalent cell types exist in the mammalian spinal cord (Becker et al., 2018) and show latent differentiation potential that can be activated in the right conditions (Llorens-Bobadilla et al., 2020; Stenudd et al., 2022). Therefore, studying the mechanisms that activate regenerative neurogenesis in fish or amphibians can inform potential therapeutic approaches to human spinal cord injury.

The generation of neurons and glia during development and adulthood is tightly controlled by factors that regulate whether NSCs remain in an undifferentiated state, enter quiescence, or differentiate. In addition to asymmetric inheritance of neuronal fate determinants (Casas Gimeno and Paridaen, 2022), other cell mechanisms control the rate of differentiation to ensure the maintenance of a pool of undifferentiated progenitors over time. For example, cell-cell signalling that represses or promotes neurogenesis can act to delineate tissue domains with different rates of differentiation (Bally-Cuif and Hammerschmidt, 2003; Janesick et al., 2015; Kicheva et al., 2014), while juxtracrine signalling (e.g., Notch signalling and downstream transcription factors) can balance the rates of differentiation over time in NSC populations (Simpson, 1990); reviewed in Henrique and Schweisguth, 2019). Additionally, microRNAs that target transcription factors and other cell fate determinants regulate both cell fate and the timing of differentiation during neurogenesis and gliogenesis (reviewed in Rajman and Schratt, 2017).

Neural progenitor cell (NPC) differentiation is regulated by transcriptional regulatory networks that integrate signals from transcription factors that either promote or inhibit differentiation. Among these, members of the basic helix-loop-helix (bHLH) transcription factor family have diverse roles: some, like the HES/Her transcription factors, act as negative regulators of differentiation by repressing proneural genes (Hu and Zou, 2022). Proneural genes, including the Neurogenin family, NeuroD/NeuroM, and Ascl1, promote cell cycle exit and neuronal differentiation (Bertrand et al., 2002; Imayoshi and Kageyama, 2014; Dennis et al., 2019). Additionally, some bHLH factors, e.g., Neurog1, suppress gliogenesis (Sun et al., 2001) or, like the Olig gene family, contribute to both neuronal and glial differentiation (Lu et al., 2001; Sugimori et al., 2008). The specific outcomes of NPC fate decisions often depend on the combinations of bHLH proteins expressed, which can either promote or inhibit distinct lineages within the nervous system (Sugimori et al., 2007). HES/Her proteins are modulated by miRNAs, short non-coding RNAs which mediate sequence-specific repression of mRNAs by binding to the 3′ untranslated regions (UTRs) of their targets (Bartel, 2018). They are predicted to be key modulators of dynamical behaviour generated by interactions within gene regulatory networks (Lai et al., 2016) and are involved in various cellular activities including CNS development and homeostasis (Saliminejad et al., 2019).

Recent evidence has revealed changes in the expression dynamics of bHLH factors as cells differentiate and points to ways in which these dynamics can enable cell fate decisions. Here, we highlight the importance of the dynamic expression of transcription factors, the role of oscillatory gene expression, and how external factors such as microRNAs fine-tune oscillations during CNS development. Additionally, we discuss the significance of these dynamic processes for the activation of quiescent NSCs. We will attempt to address this complex dynamic interaction in the context of reactivation of neurogenesis during spinal cord repair and discuss possible reasons why mammals fail to regenerate neurons.

## 2 Neurogenesis in development: the role of oscillatory gene expression

**FIGURE 1:**
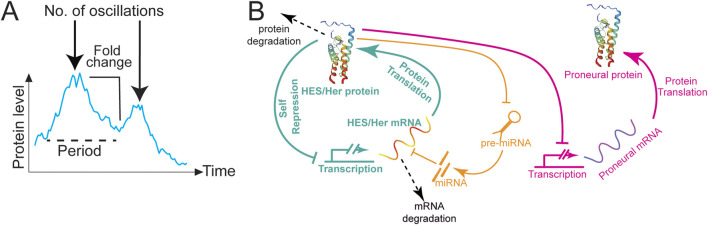
HES/Her oscillations in neural development. **(A)** Types of information encoded by oscillations. Dynamics of Her6:Venus protein expression modified from Doostdar et al. (2024). The graph depicts the intensity mean of a single cell over time (cyan line). The following dynamic parameters are highlighted: period between peaks or frequency (dashed black line); number of oscillations (black arrow); and fold-change (black line). **(B)** Schematic representation of the HES/Her autorepression network (green) including miRNA regulation (orange). HES/Her protein represses the transcription of HES/Her mRNA, leading to the periodic fluctuations in protein concentration shown in Figure 2A. miRNAs repress HES/Her mRNAs, stabilising oscillations or dampening them depending on miRNA levels. HES/Her proteins repress proneural gene expression (magenta), driving their oscillatory expression, shown in Figure 2A. Arrow lines: protein translation. T-bars: transcriptional repression. Dashed arrows: protein or mRNA degradation.

Oscillatory dynamics can encode more information than only levels, including duration (how long a signal is on), frequency, fold change, and number of oscillations (Figure 1A; reviewed in Sonnen and Aulehla, 2014), thus diversifying the potential number of responses to the same signal. The ubiquity of oscillations in biological control systems has been attributed to the fact that signalling based on detection of frequency or fold change of a signal is relatively robust to noise (Rapp, 1987; Rapp et al., 1981; Sonnen and Aulehla, 2014). Oscillatory signalling can span several orders of magnitude and can be classified according to the periodicity of the oscillations. Widely known types are infradian and circadian oscillations, with periods longer than or around 24 h, respectively (Uriu, 2016; Laje et al., 2018). In this review, we will focus on a type of oscillation known as ultradian, with periodicities of a few hours (Isomura and Kageyama, 2014; Soto et al., 2020) as they have been implicated in the control of neurogenesis during development (Kageyama et al., 2020).

Recent evidence has highlighted ultradian oscillations as a powerful mechanism that facilitates cell state transitions. This relies heavily on their ability to encode more information than just protein levels. For example, ultradian oscillators allow the cell to control the period that a regulator is on, rather than controlling the concentration of active regulator (Albeck et al., 2013) and can also provide a more robust mechanism for implementing cell-autonomous developmental timers to determine when cells differentiate (Levine et al., 2012). They can also harness stochastic processes to control the timing of differentiation events (Miller et al., 2025; Soto et al., 2020) and may be important for the coordination of signalling pathways that drive cell fate changes (Kageyama et al., 2019; Purvis et al., 2012). Lastly, different protein expression dynamics can drive different outcomes during cell state transitions (Imayoshi et al., 2013; Maeda et al., 2023; Manning et al., 2019; Marinopoulou et al., 2021; Sueda et al., 2019), which will be reviewed in more detail below.

The mechanisms behind ultradian oscillations typically involve a negative feedback loop (Goodwin, 1965; Novak and Tyson, 2008) coupled with biological time delays (Lewis, 2003; Luo et al., 2023). These delays are intrinsically linked to various cellular processes, such as transcription and translation, as well as the stability of mRNA and proteins. For ultradian oscillations to be generated by negative feedback, relatively unstable mRNA and proteins are required (Bonev et al., 2012; Hirata et al., 2002; Kiparissides et al., 2011; Tan et al., 2012), and longer mRNA half-lives may result in longer oscillation periods (Kobayashi et al., 2009). In addition, mathematical models of transcription factor oscillators predict that a sufficiently long delay in mRNA processing is required for sustained oscillations to occur (Lewis, 2003; Hirata et al., 2004; Monk, 2003b; Jensen et al., 2003). In agreement with the predictions, experimentally altering intron length of oscillatory genes involved in Notch signalling, such as *Hes7* (Harima et al., 2013), *Dll1* (Shimojo et al., 2016) and *Hes1* (Ochi et al., 2020), has been shown to influence the duration of time delays in a transcription-translation feedback loop, thus modulating the overall frequency and stability of oscillations (Ochi et al., 2020; Shimojo et al., 2016).

Among the most well-characterized ultradian oscillators expressed in NSCs and NPCs are the downstream targets of Notch signalling, Hairy and Enhancer of Split (HES/Her) transcriptional inhibitors, which are key members of the bHLH protein family expressed in neural progenitors. HES/Her proteins bind E-box DNA sequences as homo- or heterodimers and recruit co-repressors to repress target gene expression (Hu and Zou, 2022). The fact that HES/Her factors generally act as transcriptional repressors and bind their own promoter (Hirata et al., 2002) means that they can potentially drive oscillatory expression of themselves and their targets through negative autoregulation Figure 1B; (Goodwin, 1965; Novak and Tyson, 2008). Indeed, Hes1 was shown to give rise to oscillations via feedback inhibition in mice (Hirata et al., 2002; Bessho and Kageyama, 2003; Bonev et al., 2012), and this is true for related genes in other vertebrates, including zebrafish (Lewis, 2003; Giudicelli et al., 2007; Brend and Holley, 2009). The dynamic oscillation of HES/Her proteins relies heavily on the interplay between transcriptional feedback and the inherent delays that occur during mRNA processing, translation, and protein accumulation (Monk, 2003a; Jensen et al., 2003). The HES/Her negative feedback loop likely drives oscillatory expression of the proneural genes *Ngn2* and *Ascl1* Figure 1B; (Imayoshi et al., 2013; Shimojo et al., 2008; Sueda et al., 2019) and the Notch ligand *Dll1* (Shimojo et al., 2008), whereas other transcription factors, such as *Olig2,* may show HES/Her-independent oscillations (Imayoshi et al., 2013). Other ultradian oscillators are likely to be expressed in neural progenitors. However, in contrast to the presomitic mesoderm, where up to 100 cyclically expressed genes may be present in oscillatory gene networks (Krol et al., 2011), the asynchronous nature of oscillations in NSCs has precluded direct transcriptome-wide characterisation of oscillatory gene expression.

### 2.1 HES/Her ultradian oscillations in neural development

In vertebrates, HES/Her protein oscillations play crucial roles during neural development by coordinating the timing of cellular events necessary for proper tissue formation. HES/Her oscillations were initially described in somitogenesis (Aulehla and Pourquie, 2008; Cooke and Zeeman, 1976; Palmeirim et al., 1997) and more recently discovered in neurogenesis (Shimojo et al., 2008). This later discovery was because, unlike in somitogenesis, where oscillations are synchronous within the presomitic mesoderm, they tend to be asynchronous in NPCs and so detecting them requires appropriate reporters and single cell imaging (Imayoshi et al., 2015).

**FIGURE 2:**
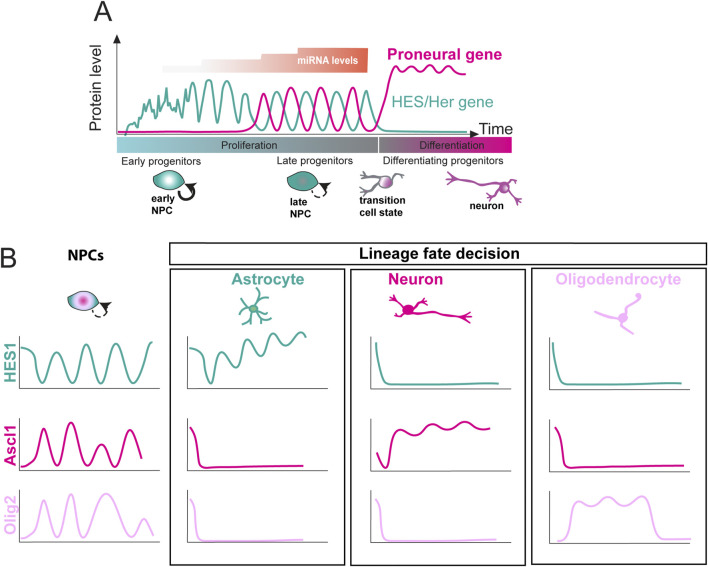
Expression dynamics of basic helix-loop-helix (bHLH) factors during neural and glial differentiation. **(A)** HES/Her and proneural protein oscillations over time during development (green and magenta lines, respectively). In early progenitors, HES/Her expression shows noisy fluctuations. miRNA levels increase over time in a stepwise manner (orange), stabilising HES/Her oscillations and reducing noise. This allows proneural gene expression, which oscillates out of phase with HES/Her genes in late progenitors. Upon differentiation, HES/Her proteins are downregulated, whereas proneural genes are upregulated with sustained dynamics. **(B)** Multiple bHLH factor cell fate determinants are co-expressed, and show oscillatory expression, in undifferentiated neural progenitor cells (NPCs). By contrast, upon commitment to a neural or glial fate, the expression of a single fate determinant becomes sustained, whereas the others are repressed. Ascl1 (magenta), Olig2 (pink), or Hes1 (green) becomes sustained in differentiating neurons (magenta), oligodendrocytes (pink), and astrocytes (green), respectively (after Kageyama et al., 2019).

The first hints of the presence of oscillations in neurogenesis came from experiments where NPCs were sorted into subpopulations of high or low HES1 expression. These cells then re-established heterogeneous protein expression levels, suggesting that high or low levels of transcription factors represented different phases of oscillations, rather than stably different expression levels in subpopulations of cells, This was supported by studies using live imaging reporters for Dll1 (a Notch ligand that promotes HES/Her protein expression) and HES1, revealing that both mRNA expression and protein fusion oscillates in the mouse embryonic neuroepithelium in a Notch-dependent manner (Figure 2A) (Shimojo et al., 2016; Shimojo et al., 2008). Furthermore, the proneural gene Neurogenin2 (NGN2) is also expressed in an oscillatory manner in neural progenitors. Inhibition of Notch signalling, a condition known to promote neuronal differentiation, leads to downregulation of HES1 and a sustained upregulation of NGN2 and Dll1. This suggests that oscillatory expression of HES1 regulates the oscillations of NGN2 and Dll1, which in turn contribute to the maintenance of neural progenitors through the mutual activation of Notch signalling (Shimojo et al., 2008). This cycling through states of high and low Notch component expression prompted researchers to ask whether gene expression oscillations have any significance for cell state transitions.

### 2.2 Ultradian oscillations and neural cell fate choice

Some cues revealing the importance of oscillatory gene expression for cell fate choice could be gleaned from observing gene expression dynamics upon neuronal or glial differentiation. Multiple cell fate determinants, such as HES1, Ascl1s and Olig2, are co-expressed in undifferentiated neural progenitors and show oscillatory expression in the undifferentiated state (Figure 2B) (Imayoshi et al., 2013; Shimojo et al., 2008). By contrast, upon differentiation into neurons, oligodendrocytes, or astrocytes, a single fate determinant is typically expressed at high levels in a sustained, non-oscillatory manner, (Ascl1, Olig2 and HES1, respectively) which precedes the upregulation of downstream factors that drive differentiation (Figure 2B) (Imayoshi et al., 2013; Shimojo et al., 2008). This suggests that different (oscillatory vs. sustained) dynamic expression of transcription factors may mediate the choice between proliferation and differentiation, with oscillatory expression of proneural genes (i.e., Ascl1) promoting proliferation, and sustained expression priming progenitors for differentiation along one of the neural or glial lineages (Sueda and Kageyama, 2020).

There is compelling functional evidence to support this theory. For example, oscillatory induction of the proneural gene Ascl1 using a light-inducible gene expression construct promotes proliferation but does not rescue neurogenesis in Ascl1-deficient progenitors, whereas sustained expression results in neuronal differentiation. Furthermore, increasing the amplitude of the Ascl1 oscillations leads to more proliferation but no differentiation, arguing that the choice between proliferation and differentiation is determined by Ascl1 expression dynamics but not absolute protein levels (Imayoshi et al., 2013). Furthermore, manipulating gene expression delays by artificially lengthening or shortening the length of Dll1 or Hes1 (a progenitor marker) genes leads to the abrogation of Hes1 oscillations in neural progenitors. Mice carrying the lengthened or shortened alleles exhibit a reduction in brain size, likely due to accelerated neural differentiation and consequent depletion of the progenitor pool (Ochi et al., 2020; Shimojo et al., 2016), arguing that oscillatory Hes1 expression is important for normal progression of neural progenitor differentiation.

Why are oscillatory dynamics important for NPC state transitions? One possibility is that the oscillatory expression of factors repressing and promoting neurogenesis allows differentiation to take place. Indeed, two recent studies examining HES/Her transcription factor oscillations in the tissue context reported that differentiation of neural progenitors is preceded by a transition from noisy, aperiodic fluctuations in gene expression levels to stable, oscillatory dynamics Figure 2A; (Manning et al., 2019; Soto et al., 2020). For example, in the zebrafish hindbrain, a transition from noisy aperiodic expression to oscillatory dynamics can be observed in single neural progenitors expressing HES1/Her6 and correlates with the peak of neurogenesis as development progresses. Furthermore, preventing this transition by specifically abolishing the regulation of *her6* by miR-9 results in a delay in neurogenesis, with cells typically being stuck in an intermediate stage of differentiation, suggesting that oscillatory transcription factor dynamics endorse the progression of cell fate decisions, and that oscillator noise is an important feature of the decoding of oscillatory expression (Soto et al., 2020).

### 2.3 miRNAs: fine-tuning ultradian oscillations during development

miRNAs can regulate their target mRNAs by inhibiting translation and/or promoting deadenylation and mRNA decay (Bagga et al., 2005; Jonas and Izaurralde, 2015; Olsen and Ambros, 1999; Standart and Jackson, 2007; Wu et al., 2006). The predominant mechanism depends on the context, with mRNA decay contributing more towards target gene repression at steady state and in post-embryonic cells (Baek et al., 2006; Hendrickson et al., 2009; Guo et al., 2010; Bazzini et al., 2012; Djuranovic et al., 2012; Eichhorn et al., 2014). Since both modes of regulation decrease protein output, regulation by miRNAs can influence the dynamics of genetic oscillators based on delayed negative feedback, including HES/Her gene oscillations (Hirata et al., 2002; Jensen et al., 2003; Lewis, 2003; Monk, 2003a).

Mathematical modelling has demonstrated that when translation inhibition and mRNA degradation are combined into a single decay parameter within a model of delayed negative feedback (autorepression), the system can produce sustained oscillations in gene expression. These oscillations occur only within a specific range of mRNA half-lives: if the mRNA is degraded too quickly or too slowly, the oscillations are dampened and eventually disappear (Xie et al., 2007), suggesting that miRNAs can influence oscillatory behaviour by modulating mRNA degradation rates.

Such regulation can cause a system to switch between oscillatory and non-oscillatory states, as demonstrated in several studies (Nandi et al., 2009; Zhou et al., 2012; Cai et al., 2013; Goodfellow et al., 2014; Li S. et al., 2016; Gao et al., 2020). For example, in nervous system development, miRNA-9 has been shown to regulate the expression of progenitor genes, including HES/Her transcription factors (Darnell et al., 2006; Leucht et al., 2008; Shibata et al., 2008; Shibata et al., 2011; Bonev et al., 2011; Coolen et al., 2012; Tan et al., 2012), as well as proneural genes (Coolen et al., 2012). Depleting or overexpressing miRNA-9 results in dampened HES1 oscillations (Bonev et al., 2012; Tan et al., 2012), supporting the notion of a “window” of miRNA concentrations that can sustain transcription factor oscillations, predicted by (Xie et al., 2007). Reciprocally, HES1 also suppresses miRNA-9 precursor expression, forming a mutual repression loop (Bonev et al., 2012). Although this can generate out-of-phase oscillations between HES1 and the miRNA-9 precursor, the mature miRNA-9 is stable and accumulates in progenitors supporting a model where rising miRNA-9 levels initially sustain HES1 oscillations, but eventually suppress them, triggering a shift to a stable HES1-low, differentiated state.

Incorporating the mutual repression motif into mathematical models introduces bistability, enabling cells to adopt either HES1-low (differentiated) or HES1-high (quiescent) states (Goodfellow et al., 2014; Castella et al., 2000; Baek et al., 2006; Sang et al., 2008; Shimojo et al., 2008; Sueda et al., 2019). Notably, while quiescent cells can resume oscillations, differentiated cells are resistant to reactivation. Thus, miRNA-mediated control of HES1 dynamics may be key to balancing progenitor maintenance, quiescence, and differentiation. Moreover, *in vivo* studies using zebrafish embryos have shown that miRNA-9 fine-tunes HES1/Her6 oscillatory dynamics by optimizing noise characteristics, enabling progenitor cells to effectively transition into differentiated neural states. This reveals a novel mechanism by which miRNAs regulate temporal gene expression patterns during development (Soto et al., 2020; Figures 1B, 2A).

In summary, ultradian oscillations are important for the progression of neural progenitors through different stages of differentiation during development and are fine-tuned by post-translational factors such as miRNAs. However, it is not clear if these tuneable dynamic processes play a role in the context of nervous system dysfunction, such as neurodegeneration or injury to the CNS. There, differentiation of resident stem or progenitor cells, or the lack thereof, can contribute to recovery outcomes. The following section will explore the significance of ultradian oscillations in regulating NSC quiescence.

## 3 Gene expression dynamics and reactivation of quiescent progenitor cells: implications for neural repair

Notch signalling is known to regulate the transitioning of neural progenitors between quiescent and proliferative states. For example, high and sustained levels of HES1 expression is associated with areas of low proliferation, such as the midbrain-hindbrain boundary (Baek et al., 2006; Hirata et al., 2001) and HES1 overexpression prolongs G-phase and attenuates proliferation (Baek et al., 2006; Maeda et al., 2023), suggesting that Notch positively regulates quiescence in mouse embryos and NSCs. On the other hand, loss of the Notch1 receptor (Ables et al., 2010) or the Notch effector Rbpj (Ehm et al., 2010; Imayoshi et al., 2010) leads to premature differentiation and consequent depletion of NSC, while deletion of Dll1 decreases the number of quiescent stem cells and increases the number of activated and differentiated NPCs in the adult mouse brain (Kawaguchi et al., 2013) suggesting that Notch is key for maintaining a reservoir of quiescent stem cells in the adult mouse. Other Notch effectors and modulators, such as non-oscillatory expression of the Notch effector Hey1, and ID proteins, have been proposed to maintain neural stem cell quiescence (Harada et al., 2021; Tzeng et al., 2001). ID proteins can dimerise with HES1, disrupting its negative autoregulatory feedback loop while preserving its ability to regulate downstream target genes, leading to suppression of neurogenesis (Bai et al., 2007).

**FIGURE 3:**
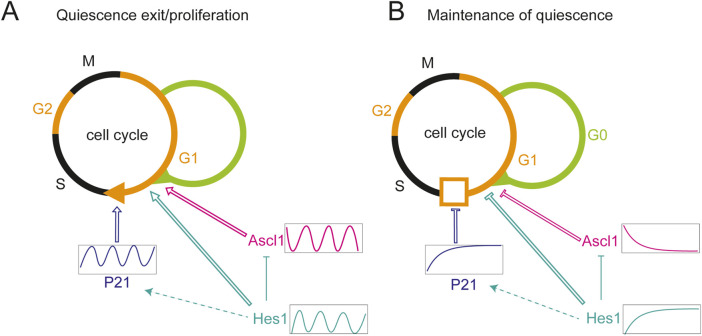
Regulation of proliferation and quiescence by HES/Her dynamics. **(A)** HES1 oscillations positively regulate proliferation by inducing oscillatory expression of P21, a negative regulator of the G1/S transition. Hes1 oscillations are permissive to quiescence exit. Oscillatory expression of Ascl1 promotes exit from quiescence, proliferation and differentiation of neural stem cells. Ascl1 oscillations may depend on Hes1 oscillations via transcriptional repression. **(B)** Sustained, high level expression of Hes1 results in high level expression of P21, inhibiting the G1/S transition. Sustained Hes1 likewise inhibits Ascl1 expression and exit from quiescence. Arrows and T-bars with open lines: activation and repression, respectively, on the level of molecular or cellular processes. Filled T-shape lines: direct repression on the molecular level. Dashed arrow: indirect activation on the molecular level. Hes1 (cyan), Ascl1 (magenta), p21 (blue).

More recently, dynamic patterns of gene expression have been implicated in regulating NPC quiescence. Oscillatory and sustained HES1 dynamics have opposite effects on cell cycle progression: oscillatory HES1 expression supports proliferation and drives oscillations of p21, a negative regulator of the G1/S transition. Sustained expression upregulates p21 expression through inhibition of ERK signalling, thus suppressing proliferation (Maeda et al., 2023). Hes1 oscillates in both quiescent and activated cells but the expression levels of oscillatory HES1 are higher in the quiescent state. By contrast, Ascl1 is not observed in quiescent cells, but shows oscillatory expression in the activated state, and inducing oscillatory Ascl1 expression leads to stem cell activation in the adult mouse brain (Sueda et al., 2019). This suggests that oscillatory Ascl1 dynamics activate quiescent neural stem cells, and high levels of HES1 inhibit reactivation by suppressing Ascl1 (Figures 3A,B).

Whilst Ascl1 oscillations can drive neural stem cell exit from quiescence, this likely depends on HES1 dynamics or protein levels. In a NSC culture model of quiescence, HES1 maintains its oscillatory expression across proliferative, quiescent, and reactivated states, indicating that oscillations are compatible with all three conditions. However, sustained HES1 expression, while supporting both proliferation and quiescence entry, prevents cells from exiting quiescence, suggesting that HES1 oscillations are required for quiescent cells to exit the dormant state (Marinopoulou et al., 2021; Figure 3B).

Emerging evidence suggests that oscillations in quiescent and proliferative NSCs also play a significant role in disease contexts. For instance, HES1 has been shown to oscillate in a proliferative breast cancer model (Sabherwal et al., 2021), Moreover, recent studies have demonstrated that HES1 dynamics changes during the dormancy and reactivation of breast cancer cells, and disruption of these oscillations prevents cell cycle re-entry and induces cell death (Cottrell et al., 2025). Interestingly, oscillatory expression of SOX2 has been observed in quiescent glioblastoma cells (Fu et al., 2024), reminiscent of HES1 oscillations in quiescent NSCs. Given that HES1 oscillations are known to facilitate the transition out of quiescence (Marinopoulou et al., 2021; Sueda et al., 2019), it is plausible to hypothesise that sustained, non-oscillatory SOX2 expression could prevent quiescent glioblastoma cells from reactivating, thereby impairing tumour reactivation and progression. Other studies have demonstrated that aged dormant NSCs, which have lost their proliferative and neurogenic potential, can regain juvenile like properties and resume functional neurogenesis following the delivery of the lentiviral vector iPaD (Kaise et al., 2022). Extraordinarily, this reprogramming induces oscillatory expression of Ascl1, a hallmark feature of active NSCs (Imayoshi et al., 2013; Sueda et al., 2019), underscoring the importance of studying of oscillation-based reactivation strategies in regenerative medicine (Kaise et al., 2022). Collectively, these findings highlight an expanding repertoire of dynamically regulated transcription factors orchestrating transitions between quiescence and proliferation in both disease and regeneration.

Another neurological condition relevant to address in the context of reactivation of neurogenesis is spinal cord injury (SCI). In mammals, it results in complete or partial paralysis due to the limited ability of the central nervous system (CNS) to repair. This is due to the lack of intrinsic capacity for axon regeneration and neurogenesis, impeding functional recovery after injury (see Table 1, for a comparison of processes occurring in non-regenerative and regenerative species) (Grossman et al., 2001). Interestingly, Notch signalling and its downstream HES/Her family transcription factors are reactivated in neural stem cells (NSCs) following SCI in both regenerative and non-regenerative species (Dias et al., 2012; Yamamoto et al., 2001). Additionally, ID genes, known positive regulators of quiescence, are triggered by injury (Tzeng et al., 2001) suggesting that ID proteins may modulate HES/Her dynamics in response to injury, thereby limiting neurogenesis and promoting the maintenance of a quiescent NSC pool. Another factor known to modulate HES/Her dynamics, named miR-9, has been implicated in the regrowth of injured axons in the larva, through repression of Her6 protein expression (Shen Y. et al., 2024). However, it remains unclear if dynamic gene expression is involved. Altogether, these findings hint that HES/Her expression dynamics (e.g., oscillatory versus non-oscillatory) may be relevant upon injury as it may influence the differentiation of resident NSCs or the lack thereof, and therefore might impact recovery outcomes. In the section below, we discuss the concept of ultradian oscillations in the context of quiescent NSC reactivation and neurogenesis during spinal cord repair.

**TABLE 1:** Differences between regenerative responses in regenerative (zebrafish, salamanders) and non-regenerative (mammals) species.

Species	Zebrafish (regenerative)	Salamanders (regenerative)	Mammals (non-regenerative)
Functional recovery	Functional recovery in 4–6 weeks following injury in the adult (Becker et al., 2004; Hui et al., 2010; van Raamsdonk et al., 1998) and within days in the larva (Briona and Dorsky, 2014; Hossainian et al., 2022; Ohnmacht et al., 2016).	Complete functional recovery in larvae and adults (Piatt, 1955; Chevallier et al., 2004; Davis et al., 1990).	Poor functional recovery (Cregg et al., 2014). A notable exception is the spiny mouse (Nogueira-Rodrigues et al., 2022)
Activation and differentiation of stem cells	Regeneration of multiple neuronal cell types from ERGs in the adult (Kuscha et al., 2012b; Reimer et al., 2008) and the larva (Briona and Dorsky, 2014; Ohnmacht et al., 2016).	ERGs proliferate and give rise to neurons during epimorphic tail regeneration (Benraiss et al., 1999).	Ependymal cells are activated upon injury but mostly generate astrocytes and, to a lesser degree, oligodendrocytes (Barnabe-Heider et al., 2010; Johansson et al., 1999; Meletis et al., 2008).
Glial scar formation and changes in ECM composition	Formation of a glial bridge that supports axonal crossing (Goldshmit et al., 2012; Mokalled et al., 2016). Deposition of pro-regenerative collagen (Wehner et al., 2017).	Proliferation of pro-regenerative glia; suppression of reactive gliosis instead of scar formation (Sabin et al., 2019).	Formation of a glial scar non-permissive to axonal regrowth (reviewed in (Bradbury and Burnside, 2019).
Capacity for axonal regeneration	Successful regeneration of spinal axons (Becker et al., 1997). Remyelination within 2 weeks (Tsata et al., 2020).	Growth of axons through the lesion site; successful regeneration (Piatt, 1955; Clarke et al., 1988; Diaz Quiroz et al., 2014). Regenerated axons myelinated within 6 weeks (Egar and Singer, 1972).	Apoptosis of neurons and glia contributing to secondary injury (Liu et al., 1997; Lou et al., 1998). Limited axonal regeneration: the environment of the lesion site is non-permissive to axonal growth, reviewed in (Zheng and Tuszynski, 2023).
Immune system response to injury	Innate immune system activation and inflammation promotes axonal regeneration and neurogenesis (Cavone et al., 2021; Ohnmacht et al., 2016; Tsarouchas et al., 2018). Signalling from macrophages and T cells promotes neurogenesis in ERGs (Cavone et al., 2021; Hui et al., 2017).	Macrophages present at lesion site (Zammit et al., 1993) Role of immune cells not known	Rapid infiltration of injury site by macrohages and neutrophils (Carlson et al., 1998; Schnell et al., 1999). Pro-inflammatory cytokines contribute to neuronal and glial death (Hermann et al., 2001; Lee et al., 2000), Reviewed in (Sterner and Sterner, 2022).
Vascular repair and angiogenesis	Rapid growth of blood vessels into lesion site preceding glial bridging and axonal regeneration. Formation of functional vessels and successful re-establishment of the blood-brain barrier (Ribeiro et al., 2023)	Not investigated	BBB breakdown and long-lasting vascular damage (Imperato-Kalmar et al., 1997; Schnell et al., 1999). Spontaneous vascular regeneration initiated but insufficient for complete repair (Figley et al., 2014; Imperato-Kalmar et al., 1997). Reactive astrocyte-dependent repair of the BBB (Faulkner et al., 2004).

## 4 Discussion

### 4.1 Reactivation of quiescent NSCs for spinal cord repair

In all vertebrate species studied so far, the adult CNS harbours NSCs that sustain constitutive neurogenesis, as well as latent NPCs that can be reactivated in response to lesions (Alunni and Bally-Cuif, 2016; Zhao et al., 2008). Nevertheless, in mammals, most of the CNS, except for NSCs of the ventricular zone (SVZ) of the lateral ventricles and the dentate gyrus of the hippocampus (Reynolds and Weiss, 1992), does not support the addition of new neurons in adulthood, and overall cellular turnover remains limited. However, quiescent cells with NSC-like properties can be isolated from various regions of the adult CNS, including the spinal cord. While these cells normally generate astrocytes and a small number of oligodendrocytes in the lesioned spinal cord, they have the potency to generate new neurons *in vitro* (Barnabe-Heider and Frisen, 2008; Barnabe-Heider et al., 2010; Deleyrolle et al., 2006; Doetsch et al., 1997; Johansson et al., 1999; Shihabuddin et al., 1997; Stenudd et al., 2022; Verma et al., 2008; Weiss et al., 1996). Their activation following injury suggests that the endogenous stem/progenitor cell niche may play a role in the limited regeneration observed after SCI (Ghosh and Hui, 2018; Vajn et al., 2013). Understanding the intrinsic properties and regenerative capacity of these endogenous glial progenitors and NSCs could significantly inform the development of SCI therapies.

**FIGURE 4:**
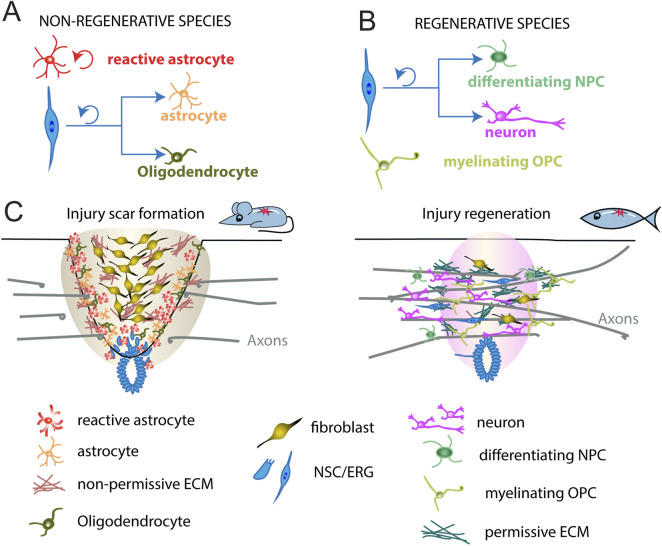
Different spinal cord repair in non-regenerative and regenerative species. **(A)** In non-regenerative animals, ependymal neural stem cells (blue) divide, leave the central canal region, and generate astrocytes (orange) and some oligodendrocytes (dark green) after spinal cord injury. Injury reactivates astrocytes to proliferate (red). **(B)** In regenerative animals, ependymo-radial cells (blue) divide, leave the central canal region, and generate new neurons (magenta) and neural progenitor cells undergoing differentiation (differentiating NPC, green). Injury reactivates OPCs to myelinate the axons undergoing repair (myelinating OPCs, light green). **(C)** In mammals, such as mouse, an injury scar is formed by invading immune cells and fibroblasts, as well as reactive astrocytes and oligodendrocytes, constituting a hostile environment to axon growth. In zebrafish, cells similarly respond to injury but, instead, generate new neurons, differentiating NPCs and myelinating OPCs to promote axonal regrowth (bridging) across the lesion site. Shown are major cell types underlying the different modes of repair in either species. NSC, neural stem cells; ERG, ependymo-radial glia. OPC, oligodendrocyte progenitor cell; NPC, neural progenitor cells; ECM, extracellular matrix.

To begin understanding the intrinsic mechanisms that promote neurogenesis after spinal cord injury, it is valuable to study regenerative species such as zebrafish and Salamanders (amniotes), and *Xenopus* larvae (Cardozo et al., 2017; Diaz Quiroz and Echeverri, 2013). Among these, zebrafish are particularly notable for their exceptional regenerative capacity, displaying full recovery of locomotor function even after complete spinal cord transection (Becker et al., 2004; Briona and Dorsky, 2014; Hossainian et al., 2022; Hui et al., 2010; Ohnmacht et al., 2016). This recovery is attributed to several key processes: the regeneration of lost neurons, generation of a permissive environment, remyelination of axons, and the re-establishment of functional neural circuits (Figure 4), further illustrated in a comparative analysis of regenerative and non-regenerative species (Table 1) (Becker et al., 2004; Becker and Becker, 2022; Becker et al., 1997; Briona and Dorsky, 2014; Cigliola et al., 2020; Mokalled et al., 2016; Tsata and Wehner, 2021; Vasudevan et al., 2021; Wehner et al., 2017).

In adult zebrafish, SCI triggers the reactivation and proliferation of progenitor cells known as ependymo-radial glia (ERGs; (Becker and Becker, 2015; Goldshmit et al., 2012; Hui et al., 2010; Reimer et al., 2009; Reimer et al., 2008). These cells possess the capacity to regenerate multiple neuronal subtypes, including motor neurons, glutamatergic interneurons, and GABAergic interneurons, within the dorso-ventral domains equivalent to those present during spinal cord development (Jessell, 2000; Becker and Becker, 2015; Becker et al., 2018; Kuscha et al., 2012b). Under normal conditions, ERGs divide slowly and primarily give rise to oligodendrocytes (Ohnmacht et al., 2016; Reimer et al., 2013; Park et al., 2007). However, following injury, they can revert to a neurogenic state (Figures 4B,C; reviewed in Becker and Becker, 2022). Similar ependymal cells are present in mammals and are triggered to proliferate after injury, but predominantly undergo gliogenesis, contributing to the formation of scar tissue (Figures 4A,C; Gregoire et al., 2015; Stenudd et al., 2015). Interestingly, unlike adults, neonatal mice exhibit enhanced spinal cord regeneration after injury, characterized by an increase in ependymal cell-like populations (Ikeda-Yorifuji et al., 2022). Similarly, juvenile mice display upregulated neuronal markers in glial cells following SCI, suggesting that regenerative neurogenesis diminishes with age and is largely lost by adulthood (Zhao et al., 2025). This divergence in injury response between species highlights the value of studying how neural stem cells (or ERGs) can be reactivated and directed toward neuronal fates following injury.

Functional studies in species that successfully regenerate spinal cord tissue after injury have identified several signalling pathways and downstream transcription factors that regulate the response of ependymal cells to injury (Table 2). This includes the reactivation of developmental signalling pathways that promote ependymal cell proliferation and neurogenesis, such as Hedgehog (Reimer et al., 2009), as well as pathways that negatively regulate both proliferation and differentiation, such as Notch (Dias et al., 2012). Presumably, the balance of these signals is important to ensure the regeneration of neurons whilst avoiding the depletion of the ependymal stem cell pool. Likewise, the transcription factor Sox2, a regulator of neural stem cell behaviour in development, is implicated in the proliferation of ependymal cells in response to injury (Fei et al., 2014; Ogai et al., 2014). Furthermore, regenerative neurogenesis in the spinal cord involves the reactivation of more than just embryonic developmental programs. For instance, macrophages recruited to the lesion site directly signal to ependymal progenitors via the cytokine Tnf-α to promote neurogenesis (Cavone et al., 2021) and neurotransmitter release following injury also regulates neurogenesis (Barreiro-Iglesias et al., 2015; Chang et al., 2021; Reimer et al., 2013). How these diverse signals converge to regulate proneural gene expression, and thereby control the rate and extent of neurogenesis, underscores the complexity of the regenerative processes making it an important area of investigation. Elucidating the dynamic gene regulatory networks that orchestrate the reactivation of neurogenesis will form the basis for uncovering the mechanisms that underlie successful regenerative outcomes.

**TABLE 2:** Signalling pathways and transcription factors implicated in regenerative neurogenesis. The following criteria were considered: (1) the availability of functional data in a regenerative species; (2) Pathway/factor is involved in regulation of neural progenitor behaviour during spinal cord regeneration (tissue regeneration or epimorphic regeneration).

Signalling molecules				
Factor/Pathway	Role	References	Model system	Type of injury
Shh	Promotes neurogenesis of motor neurons	Reimer et al. (2009)	Adult zebrafish	Transection
	Promotes proliferation of neural progenitors, regulates dorsoventral patterning	Taniguchi et al. (2014) Angell Swearer et al. (2025)	*Xenopus* larva	Tail amputation
	Promotes proliferation of blastema cells, regulates dorsoventral patterning	Schnapp et al. (2005)	Axolotl	Tail amputation
Notch	Attenuates neurogenesis	Dias et al. (2012)	Adult zebrafish	Transection
Fgf	Promotes neurogenesis and axonogenesis	Goldshmit et al. (2018)	Adult zebrafish	Transection
	Promotes proliferation and tail regeneration	Lin and Slack (2008)	*Xenopus* larva	Tail amputation
Wnt	Promotes proliferation and tail regeneration	Briona and Dorsky (2014)	Larval zebrafish	Transection
		Lin and Slack (2008)	*Xenopus* larva	Tail amputation
Tgf-beta	Promotes proliferation and tail regeneration through JunB	Ho and Whitman, 2008; Nakamura et al., 2020	*Xenopus* larva	Tail amputation
BMP	Promotes proliferation	Beck et al. (2006)	*Xenopus* larva	Tail amputation
Retinoic Acid	Promotes ependymal outgrowth and tail regeneration	Carter et al. (2011)	Newt	Tail amputation
Hippo	Promotes tail regeneration and neural progenitor survival	Hayashi et al. (2014)	*Xenopus* larva	Tail amputation
Ntf3	Promotes neurogenesis	Hui et al. (2017)	Adult zebrafish	Transection
ACh	Promotes progenitor proliferation and neurogenesis	Chang et al. (2021)	Adult zebrafish	Transection
GABA	Attenuates progenitor proliferation and neurogenesis	Chang et al. (2021)	Adult zebrafish	Transection
Dopamine	Promotes neurogenesis of motor neurons	Reimer et al. (2013)	Adult zebrafish	Transection
Serotonin	Promotes progenitor proliferation and neurogenesis of motor neurons	Barreiro-Iglesias et al. (2015)	Adult zebrafish	Transection
Tnf	Promotes neurogenesis	Cavone et al. (2021)	Larval zebrafish	Transection
dsRNA	Promotes neurogenesis	Vandestadt et al. (2021)	Larval zebrafish	Transection
P2X7	Attenuates ERG proliferation	Stefanova et al. (2024)	Adult zebrafish	Transection
Transcriptional Regulators				
Sox2	Promotes ERG proliferation	Ogai et al. (2014)	Adult zebrafish	Transection
	Supports axonal regeneration and functional recovery	Munoz et al. (2015)	*Xenopus* larva	Transection
	Promotes proliferation	Gaete et al. (2012)	*Xenopus* larva	Tail amputation
	Promotes neural stem cell proliferation	Fei et al. (2014)	Axolotl	Tail amputation
Sox11b	Promotes Ascl1 expression	Guo et al. (2011)	Adult zebrafish	Transection
AP-1 complex	Promotes neurogenesis	Cavone et al. (2021)	Larval zebrafish	Transection
AP-1 complex (JunB)	Promotes proliferation	Nakamura et al. (2020)	*Xenopus* larva	Tail amputation
	Attenuates reactive gliosis	Sabin et al. (2019)	Axolotl	Transection
AP-1 complex (JunC)	Promotes reactive gliosis	Sabin et al. (2019)	Axolotl	Transection
HDAC1	Promotes neurogenesis	Cavone et al. (2021)	Larval zebrafish	Transection
Foxm1	Promotes neuronal differentiation	Pelzer et al. (2021)	*Xenopus* larva	Tail amputation

Ultradian oscillations of HES/Her family transcription factor expression has been demonstrated to pace neurogenesis during neural development in the spinal cord (Biga et al., 2021; Manning et al., 2019). Therefore, it is plausible that precise temporal control of these oscillatory patterns is also required for effective regeneration of spinal neurons from progenitors. Notably, the decline in NSC function with age, characterized by reduced proliferative and neurogenic capacity, can be reversed, leading to the reactivation of functional neurogenesis. The underlying mechanism involves the reactivation of oscillatory Ascl1 expression, a well-established downstream target of Hes1 and a defining feature of activated NSCs (Imayoshi et al., 2013; Sueda et al., 2019), suggesting the possibility that NSC reactivation in neuronal regeneration could be driven by transcription factor oscillations (Kaise et al., 2022). Since ultradian oscillations have only recently emerged as key mechanisms involved in quiescence exit, cell fate decisions, and pathological contexts, investigating gene expression dynamics following SCI in both regenerative and non-regenerative systems could provide valuable insights. A deeper understanding of these temporal patterns may inform the development of novel therapeutic strategies aimed at enhancing spinal cord regeneration.

### 4.2 Notch signalling dynamics and the regulation of NSC quiescence: implications for spinal cord repair

Adult quiescent NSCs isolated from the mouse spinal cord retain the potential to reactivate and generate multiple cell types *in vitro* (Weiss et al., 1996). However, this potential appears to be restricted *in vivo*, and following spinal cord injury is mostly limited to the generation of astrocytes, likely due to the activation of Notch signalling in the NSC niche (Yamamoto et al., 2001). Paradoxically, Notch signalling is also upregulated in adult zebrafish following SCI (Dias et al., 2012), yet in this context, it supports successful reactivation of neurogenesis and regeneration of multiple neuronal subtypes (Kuscha et al., 2012a; Kuscha et al., 2012b; Reimer et al., 2009; Reimer et al., 2008). Therefore, details in how the Notch signalling pathway is regulated may be important for whether regenerative neurogenesis takes place. Given the established role of Notch signalling oscillations in regulating progenitor maintenance, neural cell fate decisions and NSC quiescence, it would be pertinent to investigate whether the downstream bHLH transcription factors dynamics may be facilitators of regenerative neurogenesis in species that successfully repair their spinal cords. Understanding the context dependent role of these dynamics could thus provide critical insight into why regenerative capacity is lost in mammals and how it might be therapeutically restored.

During development, the Notch target Ascl1 is required for the specification of both neurons and oligodendrocytes (Parras et al., 2004; Sugimori et al., 2008) and its expression during oligodendrogenesis is regulated by HES5 (Kondo and Raff, 2000; Liu et al., 2006), which exhibits oscillatory expression in the embryonic mouse spinal cord (Manning et al., 2019). Notably, Ascl1 itself oscillates in oligodendrocyte precursor cells, albeit at lower amplitude than in neuronal precursors. Sustained Ascl1 expression biases cell fate toward neurons at the expense of oligodendrocytes (Sueda and Kageyama, 2021). These observations suggest that Ascl1 expression dynamics, driven by HES/her dynamics, may act as a critical regulatory mechanism in determining the balance between neuronal and glial fates.

In adult mammalian NSCs, Ascl1 is required for the reactivation of quiescent cells (Andersen et al., 2014), and its oscillatory expression drives the transition from quiescence to neuronal differentiation in mice neurogenic zones (Sueda et al., 2019). This process is likely governed by HES1 oscillations (Chen et al., 1997; Imayoshi et al., 2013; Sueda et al., 2019). For example, Marinopoulou et al. (2021) showed that NSCs exhibiting sustained HES1 expression failed to exit quiescence, potentially due to the inability to reinitiate Ascl1 oscillations (Figure 3). Ascl1a expression is detected in differentiating neurons after SCI in adult zebrafish (Guo et al., 2011) while in adult rats, Ascl1 is expressed in a small number of oligodendrocyte progenitors and its overexpression stimulates the production of oligodendrocytes instead of neurons (Ohori et al., 2006). Whether differences in the expression dynamics of Ascl1 could influence differentiation outcomes upon SCI, for example by inducing a fate switch between choice of neurons or oligodendrocytes, remains to be investigated.

### 4.3 miRNAs in spinal cord repair: regulators of gene expression dynamics?

miRNAs have been implicated in the pathogenesis of spinal cord injuries (Bhalala et al., 2013), showing dysregulated expression following SCI (Li P. et al., 2016; Liu et al., 2009; Shen W. et al., 2024; Strickland et al., 2011; Tang et al., 2014; Yunta et al., 2012). Thus, manipulation of miRNAs has received considerable interest as a strategy for treating SCI (Li P. et al., 2016). There is some evidence that modulation of miRNA expression can enable the creation of a regeneration-permissive environment (Diaz Quiroz et al., 2014). For example, miRNA-9 has been implicated in the regulation of *her6* to facilitate regrowth of injured axons in the zebrafish larva (Shen Y. et al., 2024). Furthermore, it has been shown that activation of miRNA-9 leads to enhanced neuronal survival and reduced apoptosis in rodents (He et al., 2022; Wang et al., 2021) and miRNA-219, through HES5, promotes oligodendrocyte differentiation (Wang et al., 2017). Further studies have provided evidence that miRNAs regulate proneural genes. For example, in rodents, miRNA-20a and miRNA-486 are upregulated following SCI (Jee et al., 2012a; Jee et al., 2012b; Liu et al., 2009). miRNA-20a directly targets Neurogenin 1 (Ngn1), while miRNA-486 suppresses NeuroD6 leading to impaired neuronal survival and thus suggesting that their suppression would improve recovery of SCI. Since transcription factor dynamics coupled with miRNA feedback has been suggested to endow neural progenitors with the ability to enter reversible quiescence and progress to neuronal differentiation (Bonev et al., 2012; Goodfellow et al., 2014; Soto et al., 2020), it will be interesting to learn how miRNAs regulate the underlying cellular processes contributing to spinal cord repair as well as ascertain whether miRNA-regulated dynamics could influence cell fate decisions upon SCI. Some miRNAs whose function has been tested *in vivo* in animal models of SCI are listed in Table 3.

**TABLE 3:** microRNAs implicated in spinal cord repair. The following criteria were considered: (1) functional experiments carried out in a regenerative or non-regenerative species; (2) miRNA depletion or overexpression leads to a phenotype indicative of impaired or improved spinal cord repair.

microRNAs	Role	References	Species	Type of injury
let-7c	Inhibits tail regeneration	Lepp and Carlone (2015)	Newt	Tail amputation
miR-1	Inhibits tail regeneration	Lepp and Carlone (2015)	Newt	Tail amputation
miR-9	Suppresses neuronal apoptosis	He et al. (2022)	Rat	Contusion
	Promotes Mauthner axon regeneration	Shen Y. et al. (2024)	Larval zebrafish	Laser axotomy
miR-17-5p	Promotes reactive astrocyte proliferation	Hong et al. (2014)	Mouse	Partial transection
miR-20a	Represses Neurog1 and induces neurodegeneration	Jee et al. (2012b)	Mouse	Transection
miR-21	Attenuates astrocyte hypertrophy	Bhalala et al. (2012)	Mouse	Compression
miR-124	Suppresses neuronal apoptosis	Xu et al. (2019)	Mouse	Compression
miR-125b	Promotes axonal regeneration	Diaz Quiroz et al. (2014)	Axolotl	Transection
	Reduces glial scar formation	Diaz Quiroz et al. (2014)	Rat	Transection
miR-133a	Inhibits tail regeneration	Lepp and Carlone (2014)	Newt	Tail amputation
miR-133b	Promotes axonal regeneration	Yu et al. (2011)	Zebrafish	Transection
miR-145	Attenuates astrocyte hypertrophy	Wang et al. (2015)	Rat	Compression
miR-196	Regulates spinal cord patterning	Sehm et al. (2009)	Axolotl	Tail amputation
miR-200a	Promotes a NSC fate and suppresses mesodermal fate	Walker et al. (2022)	Axolotl	Transection
	Promotes glial cell proliferation, suppresses reactive gliosis and scar formation	Sabin et al. (2019)	Axolotl	Transection
miR-219	Promotes remyelination of axons	Wang et al. (2017)	Mouse	Lysolecithin-induced demyelination
miR-223	Promotes tail regeneration	Lepp and Carlone (2015)	Newt	Tail amputation
miR-486	Represses NeuroD6 and induces neurodegeneration	Jee et al. (2012a)	Mouse	Contusion

For miRNAs to be able to tune the dynamics of target gene expression, their levels must be precisely controlled. For example, too much or too little miR-9 can lead to dampening of HES1 oscillations (Bonev et al., 2012; Goodfellow et al., 2014). In hindbrain development, the levels of miR-9 increase over time in a sharp, stepwise manner, controlled spatiotemporally by the differential onset of expression of the different miR-9 precursors (Soto et al., 2022). However, the mechanisms that regulate the transcriptional expression of miRNA precursors in response to SCI remain unknown. In adult vertebrates, miRNA can also be delivered to the injury site through cerebrospinal fluid encapsulated in exosomes. This exosomal delivery of miRNAs has been extensively studied as a potential therapeutic strategy for promoting recovery after SCI. Yet, the levels and timing of delivery for optimal therapeutic effect needs to be addressed (Pan et al., 2021; Feng et al., 2021).

In summary, factors that modulate bHLH transcriptional oscillations are implicated in the regulation of NSC quiescence and are also expressed in the context of central nervous system (CNS) injury. These observations provide hints of the potential involvement of dynamic gene expression in neural regeneration. Based on recent studies demonstrating a role for oscillatory gene expression dynamics in the reactivation of stem cells (Cottrell et al., 2025; Kaise et al., 2022; Marinopoulou et al., 2021), we hypothesise that, during spinal cord repair, dynamic gene expression is necessary for cells to make cell fate decisions precisely orchestrated in time and space. Further, we hypothesise that these dynamics are fine-tuned by microRNAs, which may influence cellular differentiation outcomes following spinal cord injury. So far, the role of HES/Her oscillatory dynamics in spinal cord repair remains poorly understood, primarily due to the lack of experimental approaches for intravital imaging of dynamic gene expression in adult animals.

In the following section, we will explore current strategies for detecting and manipulating gene expression dynamics in both regenerative and non-regenerative contexts.

### 4.4 Potential approaches to the study of gene expression dynamics during spinal cord repair

Understanding ultradian oscillations of transcription factors has gained increasing attention over the past 2 decades, closely paralleling the development of techniques for visualizing gene expression dynamics in living organisms. Despite these advances, visualizing ultradian oscillations in the CNS in whole organisms remains a significant challenge. The development of tools to label endogenous proteins has become more accessible with the advent of CRISPR-Cas technology and the generation of knock-in models. However, live imaging remains a major hurdle. To date, most studies have been conducted in isolated NSCs, NPCs, *ex vivo* mouse tissue sections, or *in vivo* in zebrafish embryos and larvae. Due to the complex tissue context of nervous system regeneration, cell culture or tissue slice models are not appropriate for addressing gene expression dynamics in these conditions. Therefore, moving the field forward, particularly in the context of spinal cord injury, will require further innovation to enable such studies in adult organisms.

Recent advances in imaging techniques offer promising directions. For example, in adult zebrafish, the application of multiphoton microscopy, specifically intravital three-photon microscopy (3PM), has allowed deep-tissue imaging up to 300 µm with subcellular resolution in the adult brain of albino zebrafish (Choe et al., 2022; Hontani et al., 2022). In mammals, however, reduced tissue transparency presents an additional challenge. Because mammalian tissues are relatively transparent to near-infrared (NIR) light, the use of NIR fluorescent proteins (NIR-FPs) in combination with 3PM could enable non-invasive imaging of tissues at depths of up to 3 mm (Shcherbakova et al., 2018) which has already been demonstrated using two-photon microscopy (2PM) (Tong et al., 2021).

Moreover, the use of implanted glass windows in combination with advanced imaging could revolutionize the ability to monitor ultradian oscillations in the CNS (Fenrich et al., 2013). Nevertheless, a major obstacle remains: ultradian oscillations occur on timescales of minutes to hours, necessitating high-frequency, long-duration intravital imaging. To address this, artificial intelligence (AI) and mathematical modelling can be employed to infer dynamic gene expression between imaging time points. For example, the OscoNet framework can infer transcriptional oscillators from single-cell RNA sequencing data (Cutillo et al., 2020). Finally, to not only observe but also manipulate gene expression dynamics in adult animals, optogenetic approaches are rapidly advancing and may offer powerful tools to induce controlled oscillations *in vivo* (Riefolo et al., 2019; Sanchez-Sanchez et al., 2025).

In summary, miRNA-modulated ultradian oscillations in transcription factor expression are essential for normal development of the nervous system, allowing transitions between quiescence, proliferating and differentiating states. Notch signalling and the ultradian dynamics of its downstream effectors have emerged as key regulators of stem cell quiescence in the adult CNS. However, the significance of these dynamics for nervous system repair in response to injury remains unclear, and addressing this question will require live imaging of dynamics in the native tissue context. While intravital imaging of ultradian oscillations remains a formidable challenge, emerging technologies, including high-resolution microscopy, photo-switchable proteins, and AI-driven analysis, are rapidly evolving and promise to significantly advance our understanding of spinal cord repair and dynamic gene regulation.
